# Annotations

**Published:** 1895-01-26

**Authors:** 


### ANNOTATIONS.

### Marriage, or not Marriage, for Women?

DR. HENRIETTA KEATING affirms that though "marriage may be a masquerade to some, and a speculation to others," yet "every marriage affects the world for good or evil, and its influence is eternal." The expressions used perhaps savour more of the expansiveness of poetry than of the precision of science; nevertheless, it sometimes happens that undefined expansiveness is more truly and largely scientific than definite precision. The specialty of the woman, to speak in the scientific jargon of the period, is marriage. It may well be said that normal woman finds her natural contentment and life-work in marriage alone. In our soberest judgment, the civilisation which fails to encourage marriage, and to find suitable house-mates for the great mass of its healthy women of every class is a failure. If it be true that "man cannot live by bread alone," it is equally true that man cannot live by "brains" alone. Much less can woman. The things of the mere intellect, and of the artistic faculties, endure for a moment; at most for a few brief years of a woman's earlier and less experienced period of life; but the things of the affections, of the heart, of the deeper soul; the love of husband and of children—these endure for ever. No statesman could better serve his country and civilisation generally, than by endeavouring to make the conditions of life easier and the struggle less keen, and thus encouraging fairly early marriage among those best fitted for it.

### A Song or a Sermon?

MR. A. J. BALFOUR addressed the members of the Manchester and Salford Equitable Co-operative Society last week, and at the outset of his speech expressed the conviction that most of his hearers would prefer a good song to a speech, even on their own favourite topic of co-operation. There are three classes of persons concerned with physic who would be the better for a little of the genial worldliness which prefers at proper times a song to a sermon. These three classes are doctors, nurses, and patients. For preternatural solemnity which cannot laugh, and which sees something unscientific in the mere suspicion of a joke, commend us to the members of the modern medical profession. No other profession can equal our own for mental and moral severity, unless, indeed, it be the confraternity of sick-nurses. Now this is a question of a good deal of importance to the third class of persons of whom we are speaking, that is, to our patients. In the matter of cheerfulness, the doctor may kill and the doctor may cure. Probably few of us suspect how very delicate the balance sometimes is on which life and death are turning. An hour's depression of spirits after the visit of a too portentous physician may determine the dip of the scale the wrong way. On the other hand, the bright and hopeful visit of a cheery doctor may not only do good for hours afterwards, but the daily anticipation of such a visit may carry a man victoriously through in cases where no other medicine whatsoever would produce the same result. Modern medical science tends a little towards medical pessimism by showing us with such startling distinctness the manifold possibilities of danger which are associated with almost every morbid condition; and this deep knowledge unconsciously deepens the gravity of our apprehensions, and clouds our faces with injurious doubts. But in medicine, as in other things, it is often the best philosophy to "live by the day." The ghosts of possible dangers which may be present in our own minds ought to be resolutely "laid" in the presence of our patients. In every case it is not only our duty, but should be our pleasure, to present the very best possible prognosis. The bedside is not the place for a critical balancing of chances, and the patient is not the person, neither, indeed, is the nurse, with whom such a discussion should be carried on. Both doctor and nurse presumably have but one end in view—the prompt cure of the patient; and to this end there is, perhaps, no single factor of more importance than a cheerful and hopeful moral atmosphere all the day long.

### Is London Healthy?

DEATH is always busy among the young and among the old, but there are ages which are largely spared, and thus it happens that the proportion of people of the different ages in any town makes all the difference in regard to the meaning of its death-rate. The proportion of the sexes at the different ages also has an influence; and it is only since the Registrar-General made these corrections for peculiarities of age and sex distribution in London and the large towns that any proper comparison has been possible. In his last report the medical officer to the County Council has given the age and sex distribution in the different and various London districts, by aid of which Dr. Vivian Poore has been enabled to compare their corrected death-rates with those of the country at large. These results are sufficiently startling. They show the enormous mortality of the central districts, and are certainly enough to make any one pause before repeating the parrot-cry that London is a healthy dwelling-place. Taking the difference between the "standard" death-rate, or what it should be according to the age and sex distribution, and the "corrected" death-rate, or what it really is, we find that only in Plumstead, Hampstead, Lewisham, Wandsworth, and Hackney is the corrected death-rate less than the average standard for England and Wales, and that these healthy districts only contain altogether 659,000 out of the four millions of people living in this London of ours. Everywhere else the death-rate is above the standard, creeping up from Paddington, St. George's, Hanover Square, and Kensington, where the excess is only 2·02 on a rate of 17·38 per thousand, to the enormous excess in the Strand of 17·16 on a standard rate of 16·24; that is to say that in one central district in London the people die, age for age and sex for sex, at more than twice the rate they do in the whole of England and Wales, even after including within England and Wales London and all the large towns. The answer, then, to our question is that, although certain outlying districts are in a very good condition, central London, the part which is commonly known as London by the outside world, is in a parlous plight, reflecting but small credit upon those who built it, and giving but short shrift to those who dwell therein.

